# Bioaccumulation and toxicity of selenium compounds in the green alga *Scenedesmus quadricauda*

**DOI:** 10.1186/1471-2229-9-58

**Published:** 2009-05-15

**Authors:** Dáša Umysová, Milada Vítová, Irena Doušková, Kateřina Bišová, Monika Hlavová, Mária Čížková, Jiří Machát, Jiří Doucha, Vilém Zachleder

**Affiliations:** 1 Laboratory of Cell Cycles of Algae, Division of Autotrophic Microorganisms, Institute of Microbiology, Academy of Sciences of the Czech Republic, 379 81 Třeboň, Czech Republic; 2 Research Centre for Environmental Chemistry and Ecotoxicology – RECETOX, Faculty of Science, Masaryk University, 625 00 Brno, Czech Republic

## Abstract

**Background:**

Selenium is a trace element performing important biological functions in many organisms including humans. It usually affects organisms in a strictly dosage-dependent manner being essential at low and toxic at higher concentrations. The impact of selenium on mammalian and land plant cells has been quite extensively studied. Information about algal cells is rare despite of the fact that they could produce selenium enriched biomass for biotechnology purposes.

**Results:**

We studied the impact of selenium compounds on the green chlorococcal alga *Scenedesmus quadricauda*. Both the dose and chemical forms of Se were critical factors in the cellular response. Se toxicity increased in cultures grown under sulfur deficient conditions. We selected three strains of *Scenedesmus quadricauda *specifically resistant to high concentrations of inorganic selenium added as selenite (Na_2_SeO_3_) – strain SeIV, selenate (Na_2_SeO_4_) – strain SeVI or both – strain SeIV+VI. The total amount of Se and selenomethionine in biomass increased with increasing concentration of Se in the culturing media. The selenomethionine made up 30–40% of the total Se in biomass. In both the wild type and Se-resistant strains, the activity of thioredoxin reductase, increased rapidly in the presence of the form of selenium for which the given algal strain was not resistant.

**Conclusion:**

The selenium effect on the green alga *Scenedesmus quadricauda *was not only dose dependent, but the chemical form of the element was also crucial. With sulfur deficiency, the selenium toxicity increases, indicating interference of Se with sulfur metabolism. The amount of selenium and SeMet in algal biomass was dependent on both the type of compound and its dose. The activity of thioredoxin reductase was affected by selenium treatment in dose-dependent and toxic-dependent manner. The findings implied that the increase in TR activity in algal cells was a stress response to selenium cytotoxicity. Our study provides a new insight into the impact of selenium on green algae, especially with regard to its toxicity and bioaccumulation.

## Background

Selenium is a trace element, which affects organisms in a dose-dependent manner. At low levels, it contributes to normal cell growth and function. It has a anti-carcinogenic effect [1-3], plays a role in mammalian development [4], immune function [5], and in slowing down aging [6]. On the other hand, high concentrations are toxic, causing the generation of reactive oxygen species (ROS), which can induce DNA oxidation, DNA double-strand breaks and cell death [7].

In algae, the essentiality of selenium has been studied mainly in marine species. Selenite bioconcentration by phytoplankton [8] and selenium requirements of many of phytoplankton species from various taxons was demonstrated [9]. Unicellular, marine calcifying alga *Emiliania huxleyi *requires nanomolar levels of selenium for growth and selenite ion is the predominant species used by this alga [10]. Se is essential to many algae [11] including *Chlamydomonas reinhardtii *[12]. The essentiality, however, is sometimes difficult to estimate because selenium is required at such low levels for most organisms that it is experimentally challenging to generate strong phenotypes of deficiency [13].

The function of selenium is mediated mostly by selenoproteins, to which the selenium as a selenocysteine is inserted during translation [14,15]. Selenoproteins include enzymes such as glutathione peroxidases (GPx), thioredoxin reductases (TR), proteins implicated in the selenium transport (selenoprotein P) and proteins with unknown functions, which are involved in maintaining the cell redox potential [15].

Most of the selenoproteins are found as animal proteins. They have not been found in yeast and land plants. Surprisingly, they have been detected in the green alga *Chlamydomonas reinhardtii. Chlamydomonas *uses selenoenzymes and the repertoire is almost comparable to that in mammalian models [16]. A survey of the Chlamydomonas genome led to the identification of the complete selenoproteome defined by 12 selenoproteins representing 10 families [17,18]. The unicellular alga *Ostreococcus *(Prasinophyceae) and ultra small unicellular red alga *Cyanidioschyzon *(Cyanidiaceae) also use selenoenzymes [19-21] as well as *Emiliania huxleyi *(Haptophytes) [22]. Among these selenoenzymes, one of the form of thioredoxin reductase (TR) was also identified [16]. The thioredoxin system, comprising thioredoxin (TRX), TR and NADPH works as a general protein reductase system [23].

In the cytosol and the mitochondria, thioredoxins are reduced by NADPH through the NADPH thioredoxin reductase (NTR) present in these compartments. NTR is universally distributed from bacteria to mammals, but two different forms have evolved. The first corresponds to a low molecular weight NTR found in bacteria, yeast, and plants. Mammals contain a distinct form of NTR, which contains selenocysteine [24].

Of the 4 NTRs found in *Chlamydomonas*, one of them was quite unexpected since it is a mammalian type NTR containing a selenocysteine residue [15,16]. This NTR is also encoded in another alga, *Ostreococcus*, but not in land plants [25]. Some authors showed that TR provides active selenide for the synthesis of selenoproteins and is an important protector of cells against Se toxicity [26-28].

Besides the presence of selenium in selenocysteine, selenium can substitute sulfur in methionine and form selenomethionine. This can be incorporated nonspecifically into proteins instead of methionine. This misincorporation may result in significant alterations in protein structure and consequently protein function causing a toxic effect of Se in land plants [29].

In model algal organisms, studies of the effects of both selenite and selenate on the green alga *Chlamydomonas reinhardtii *showed ultrastructural damage to chloroplasts resulting in impaired photosynthesis [30,31]. In *C. reinhardtii *selenite is transported by a specific rapidly saturated system at low concentrations and non-specifically at higher concentrations [32]. Fluxes for selenite uptake were constant, while fluxes for selenate and SeMet uptake decreased with increasing concentrations, suggesting a saturated transport system at high concentrations [32]. In *Scenedesmus obliquus*, phosphate enrichment leads to considerable decrease of Se accumulation [33]. In *Chlorella zofingiensis *the accumulation of boiling-stable proteins and the increased activities of the antioxidant enzymes suggested that these compounds were involved in the mechanisms of selenium tolerance [34].

Here, we studied the response of the wild type of the green alga *Scenedesmus quadricauda *and its three selected strains to the presence of selenite and selenate of different concentrations. Strains were selected to be resistant to high doses of selenite or selenate or both. To monitor cellular response, we followed the growth rate, the total amount of Se and selenomethionine in algal biomass and the activity of thioredoxin reductase. The effect of the presence of selenium compounds in cultures deprived of sulfur was also studied.

## Results and discussion

### Toxicity of selenium and selection of selenium resistant strains

**Figure 1:**
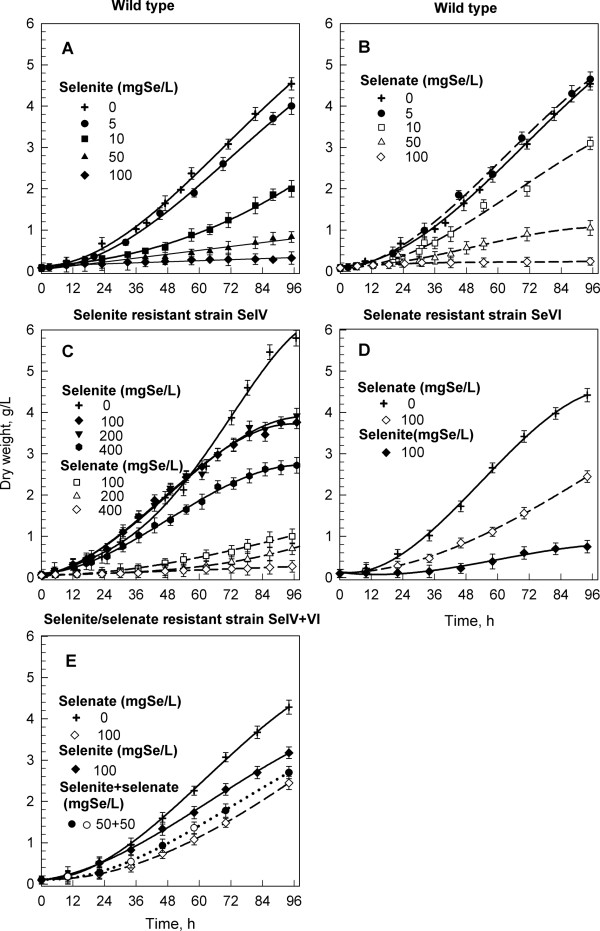
**Effect of different selenium concentrations on the growth of *Scenedesmus quadricauda***. Effect of different concentrations of selenite or selenate on the growth of the wild type (**A**, **B**), selenite resistant strain SeIV (**C**), selenate resistant strain SeVI (**D**) and selenite/selenate resistant strain SeIV+VI (**E**) of *Scenedesmus quadricauda*. Data are presented as means ± S.D. of triplicate experiments.

**Figure 2:**
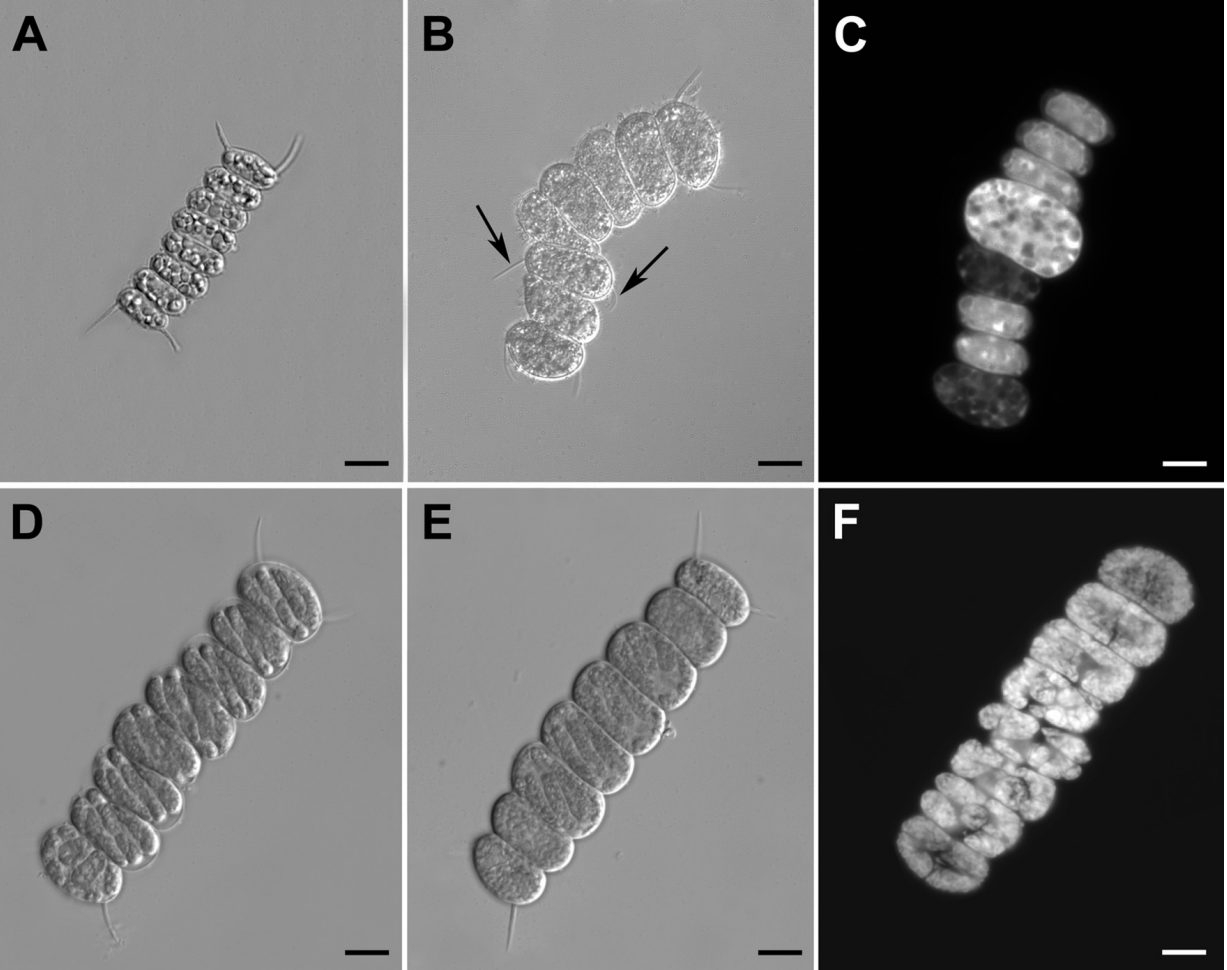
**Microphotographs of eight-celled coenobia of the wild type and Se resistant strains of *Scenedesmus quadricauda *treated with selenium**. Coenobia observed in DIC (**A**, **B**, **D**, **E**) or in a fluorescence microscope (**C**, **F**). **A**: daughter untreated cells in octuplet coenobium; **B**: cells treated with selenite 50 mg Se × l^-1^, malformations of the cells and an abnormal number of spines (see arrows) are apparent; **C**: cells treated with selenite 100 mg Se × l^-1^, only one large bright cell from the coenobium was viable but not dividing, five small half-bright cells were growing poorly and two dark cells were dead. **D**, **E**, **F**: the cells in octuplet coenobium at the stage of protoplast division, **D**: selenate resistant strain SeVI treated with selenate 100 mg Se × l^-1^, **E**: selenite/selenate resistant strain SeIV+VI treated with selenite+selenate (50+50 mg Se × l^-1^), **F**: selenite resistant strain SeIV treated with selenite 100 mg Se × l^-1^, bars: 10 μm.

Cells of the wild type strain of *Sc. quadricauda *were grown in the presence of selenite or/and selenate at concentrations from 0 to 100 mg Se × l^-1 ^(Figures 1A and 1B). At Se concentrations > 50 mg Se × l^-1 ^most of the cells died within one or two days of culturing. At Se concentrations <= 10 mg Se × l^-1 ^the cells were able to grow although the growth rate was diminished in a dosage proportional way (Figures 1A and 1B). Selenite showed a higher toxic effect than selenate. Already a concentration of 10 mg Se × l^-1 ^of selenium as selenite slowed the growth rate drastically (compare Figures 1A and 1B). Microscopic observations showed that the number of dead cells increased progressively with increasing concentration of selenite. Poisoning by selenium caused bleaching of chloroplasts, cell malformations, e.g. increased number of spines (Figure 2B) and finally, cell death. A very small fraction of cells (< 1%), however, remained viable. At least for several days they grew but did not divide and also died in the end (Figure 2C). Some of these cells were able to recover if transferred into selenium free nutrient solution. Thereafter, the recovered cells showed a higher resistance to selenite than the wild type cells. By repeating this procedure, we finally selected those cells, which were able to grow in extremely high concentrations of selenium (up to 400 mg Se × l^-1^) if added in the form of selenite (Figures 1C and 2F). Their growth rate was even higher than in the untreated wild type. Although the strain was resistant to the high levels of selenite, its sensitivity to selenate was comparable to that of the wild type (Figure 1C). Therefore, by using the same procedure, we have attempted to select a strain resistant to high levels of selenate. While the resistance to high levels of selenate was successfully attained the strain remained sensitive to high levels of selenite (Figures 1D and 2D). Finally, we selected the strain able to grow both on selenite and selenate (Figures 1E and 2E). This strain was more resistant than the wild type, however, more sensitive to both compounds than the respective resistant strains (compare Figures 1C, D and 1E). Due to possible use of these strains both as a nutritional supplement for animals or humans and for land remediation the strains were patented [35-37].

In contrast to *Scenedesmus*, no adaptation mechanisms were observed in *Chlamydomonas*. The authors found that chloroplasts were the first target of selenite cytotoxicity, with effects on the stroma, thylakoids and pyrenoids. At higher concentrations, they observed an increase in the number and volume of starch grains and electron-dense granules containing selenium [31].

The present findings confirmed the diverse effect of selenite and selenate on the cells, which is probably caused by distinct mechanisms of uptake and further metabolisms of different Se compounds as found in land plants and Cyanobacteria [38,39]. Selenate is accumulated in land plant cells against its likely electrochemical potential gradient through a process of active transport [29]. Selenate readily competes with the uptake of sulfate and it has been proposed that both anions are taken up via a sulfate transporter in the root plasma membrane in land plants. Selenate uptake in other organisms, including *Escherichia coli *[40], yeast [41] and *Synechocystis sp*. [38] is also mediated by a sulfate transporter [39].

Selenite uptake was significantly lower than selenate uptake in willow [42]. However, the sensitivity of algae to the element has been shown to be highly species-dependent. For instance, it was found that concentrations of selenate inhibiting growth could vary as much as three orders of magnitude depending on the species tested [43]. Moreover, natural phytoplankton communities could be more sensitive than single species, grown in optimal conditions in the laboratory [44].

Unlike selenate, there was no evidence that the uptake of selenite is mediated by membrane transporters. The mechanism of selenite uptake by plants remains unclear. Recently, selenite uptake in wheat has been found to be an active process likely mediated, at least partly, by phosphate transporters. Selenite and selenate differ greatly in the ease of assimilation and xylem transport [45]. Selenate assimilation follows, in principle, that of sulfate and leads to the formation of SeCys and SeMet. Selenite is reduced to selenide and then forms selenoaminoacids [46].

We found that selenite was more toxic than selenate and caused more severe growth inhibition, which is in line with findings in land plants. This might be due to the faster conversion of selenite to selenoaminoacids in the species studied [47]. On the other hand, selenate was reported to be more toxic than selenite and caused more severe growth inhibition in grass species [48].

### Growth of sulfur deficient cells in the presence of selenite

*Chlamydomonas *growth does not appear to depend on added Se, presumably because sufficient Se is present as a trace contaminant in other media components. However, it is conceivable that the demand for Se increases under stress conditions where redox metabolism and hence participation of selenoproteins is stimulated [24]. We have found a low but easily measurable amount of selenium in cells grown in medium without added selenium compounds and in which the selenium intracellular amount increased when the sulfur level was low (Table 1). Testing the assumption that the cells have a trace amount of selenium even in "selenium free" medium, we found that in the MgSO_4 _used as a source of sulfate and magnesium for a nutrient medium (Lachner, p.a., Penta, p.a), Se was, indeed, present in a range from 0.1 to 0.2 mg × kg^-1^.

**Table 1:** Selenium and sulfur content in biomass of *Scenedesmus quadricauda*

	**Selenite **mg Se × l^-1^	**0**			**10**			**50**	
	
**Nutrient solution**	**Sulfate **mM	**400**	**40**	**4**	**400**	**40**	**4**	**400**	**40**
**Cells**	**Selenium **mg/kg D.W.	1.2	0.8	13.2	706	678	689	3500	3730
	
	**Sulfu**r mg/kg D.W.	3300	3985	890	4240	4640	230	4240	4120

Selenium and sulfur content in biomass of the wild type of *Scenedesmus quadricauda *grown in nutrient solution with sulfate concentrations 400, 40, and 4 mM in the absence or the presence of selenite at concentrations 10 or 50 mg Se × l^-1^.

**Figure 3:**
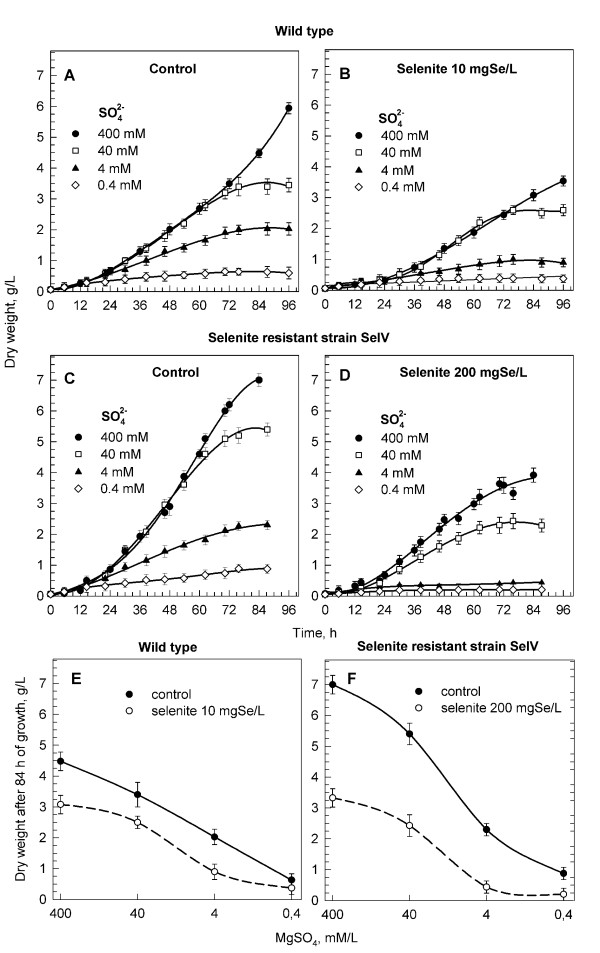
**Growth of *Scenedesmus quadricauda *in nutrient solutions with different sulfate and selenite concentrations**. Growth of *Scenedesmus quadricauda *wild type and selenite resistant strain SeIV in nutrient solutions with different sulfate concentrations in the absence (**A**, **C**) or the presence of selenite (**B**, **D**). Concentrations were chosen to be of low toxicity for wild type and strain SeIV (10 and 200 mg Se × l^-1 ^selenite respectively). **E**, **F**: dry weight (g × l^-1^) attained in cells of a wild type (**E**) and selenite resistant strain (**F**) after 84 hrs of growth in the absence and presence of selenite. Data are presented as means ± S.D. of triplicate experiments.

Asynchronous populations of the wild type and selenite resistant cells (strain SeIV) were grown in concentrations 0.4, 4, 40, 400 mM sulfate in a nutrient medium in the presence or absence of selenite. The concentrations 10 mg Se × l^-1 ^and 200 mg Se × l^-1 ^of selenite were added to the wild type and strain SeIV respectively. These concentrations were known to be well tolerated for the tested strains. As can be seen in Figure 3, both strains were affected by sulfur deficiency in the same way. No effect on growth rate occurred at sulfate concentrations >= 40 mM, but cells at lower sulfate concentrations entered a stationary phase earlier (at ca. 72 h of growth) (Figures 3A and 3C). The total sulfur content in the wild type biomass grown at 400 and 40 mM sulfate was comparable as 40 mM was a sufficient amount to keep cells growing well at least for 72 hours (Table 1).

With a further decrease of sulfur concentrations (4 mM and 0.4 mM), the growth rate of cells as well as the interval of growth progressively decreased (Figures 3A and 3C). The total sulfur content in biomass also decreased; it was not even possible to obtain an appropriate amount of biomass for analyses at 0.4 mM sulfate, as the culture grew so poorly (Table 1).

The growth of sulfur deficient cells in the presence of selenite was more affected than in its absence both in the wild type (Figures 3B and 3E) and selenite resistant strain (Figures 3D and 3F). The total selenium content in biomass was, however, independent of sulfate concentration and was proportional to selenium concentration in the nutrient solution (Table 1).

The increasing selenium toxicity with sulfur deficiency indicates interference of Se with sulfur metabolism, possibly resulting from non-specific replacement of sulfur by selenium in proteins and other sulfur compounds. In land plants, Se toxicity is associated with the incorporation of selenocystein (SeCys) and selenomethionine (SeMet) into proteins in place of Cys and Met. The differences in size and ionization properties of S and Se may result in significant alterations in structure and consequently function of proteins [39].

### Amount of intracellular selenium and selenomethionine

**Figure 4:**
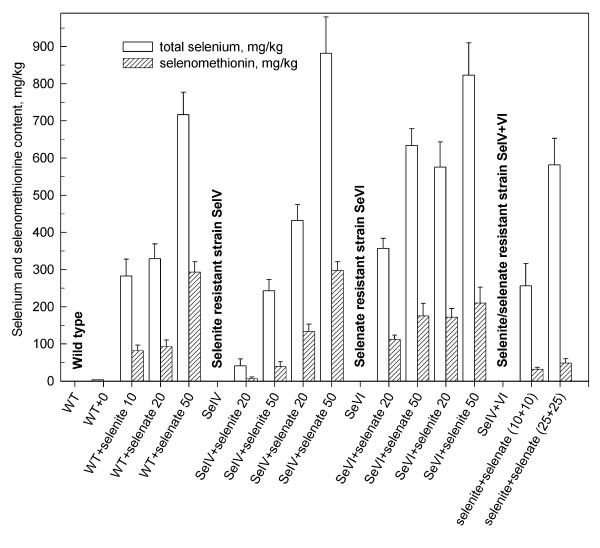
**Total selenium and selenomethionine content of dried biomass of *Scenedesmus quadricauda***. Total selenium and selenomethionine content in mg per kg of dried biomass of the wild type and selenium resistant strains of *Scenedesmus quadricauda *grown at concentrations of selenite or selenate (0, 20, 50 mg Se × l^-1^). WT: wild type; SeIV: selenite resistant strain; SeVI: selenate resistant strain; SeIV+VI: selenite/selenate resistant strain. White bars: total selenium content, dashed bars: selenomethionine content. Data are presented as means ± S.D. of triplicate experiments.

Using ICP-MS, the total amount of Se compounds was determined in both the wild type and Se resistant strains to which selenium had been added as selenite or selenate or mixture of both 20 and 50 mg Se × l^-1 ^(Figure 4). A value of 10 mg Se × l^-1 ^in the case of selenite was chosen since, due to its toxicity, the cells of wild type died very early at higher concentrations of selenite, making it impossible to obtain sufficient biomass to perform the necessary analyses. In the case of the strain tolerant to both selenite and selenate, the selected concentrations were such that the cell obtained the identical amount of selenium (20 and 50) in sum as the wild type. In addition, the amount of selenomethionine was determined separately. Table 2 shows the % of total Se (SeMet) for all cases shown in Figure 4.

**Table 2:** Percentage of selenomethionine in a total cellular Se in wild (WT) and selenium resistant strains (SeIV, SeVI, SeIV+VI) of *Scenedesmus quadricauda *grown in the presence of selenite or selenate

**Se compound** **added**	**Se mg × kg**^-1^ **added**	**%SeMet** **of cellular Se**
***WT***		

0	0	0.00

selenite	10	28.87

selenate	20	28,24

selenate	50	40.86

**SeIV**		

selenite	20	17.00

selenite	50	16,05

selenate	20	31.02

selenate	50	33.79

***SeVI***		

selenate	20	31.37

selenate	50	27.60

selenite	20	29.86

selenite	50	26.00

**SeIV+VI**		

selenite+selenate	20 (10+10)	12.50

selenite+selenate	50 (25+25)	8.32

All strains grown in the absence of selenium possessed a very low amount of intracellular Se and SeMet. Increasing the Se concentration added both in form of selenite and selenate caused a dose-dependent increase of the total content of Se and SeMet in the wild type. In the presence of selenate 50 mg Se × l^-1 ^in media, the SeMet content reached 300 mg × kg^-1^.

In the selenite resistant strain SeIV, the total Se content and SeMet was low (20 – 40 mg × kg^-1^) in the presence of selenite. In contrast, the presence of selenate caused the total Se content to increase markedly above 850 mg × kg^-1^and was even higher than in the wild type. The finding that the SeIV strain treated with selenite has much lower levels of total Se and SeMet shows that its tolerance mechanism is probably exclusion. Its Se and SeMet levels are similar to the wild type when treated with selenate, explaining its lack of selenate tolerance and also showing that selenate and selenite are imported in this alga by different mechanisms.

In the selenate resistant strain SeVI, the presence of selenate caused a moderate increase in Se (up to 600 mg × kg^-1^) and SeMet content (up to 160 mg × kg^-1^). The presence of selenite increased the Se (800 mg × kg^-1^) and SeMet (210 mg × kg^-1^) content markedly. The SeVI strain shows no difference from WT in terms of total Se and SeMet levels, indicating that its tolerance mechanism is not exclusion but must be something internal, a way to detoxify or sequester the Se intracellularly.

The double-tolerant strain (SeIV+VI) has exceptionally low SeMet fractions (up to 50 mg × kg^-1^) compared to the other strains, which could indicate a change in Se metabolism, perhaps reduced assimilation from inorganic to organic Se.

Our results indicate that the increase of SeMet amount in the cells was correlated to toxicity of a given type of the added inorganic Se compound. The amount of selenium and SeMet in algal biomass was, in addition to its dependence on the type of the compound, also dose-dependent (compare bars of 20 and 50 mg Se × l^-1 ^in Figure 4).

Papers dealing with the identification of selenium compounds in algae biomass are less frequent than those dealing with other systems. Several selenium compounds (dimethylselenopropionate, Se-allylselenocysteine, selenomethionine) were identified in the green alga *Chlorella vulgaris *[49]. Selenomethionine was present only in ng × g^-1 ^concentrations. In *Chlorella *treated with selenate and selenite the content of selenomethionine was determined using K-edge X-ray absorption spectroscopy [50]. It comprised 39% and 24% of the accumulated Se when treated with selenite and selenate respectively. An effort to quantify Se compounds (fractionation) can be found in [15] dealing with selenized blue-green alga *Spirulina platensis*. Cultivation with selenite up to 40 mg Se × l^-1 ^stimulated the growth of *Spirulina*. It was demonstrated that inorganic selenite could be transformed into organic forms. The organic selenium accounted for 85.1% of the total accumulated selenium and was comprised of 25.2% water-soluble protein-bound Se.

According to our results, the SeMet content (29% and 41%) in *Scenedesmus quadricauda *after incubation with selenite and selenate, respectively was comparable to the results obtained in *Chlorella *(24% and 39%) [50].

### Activity of thioredoxin reductase

**Figure 5:**
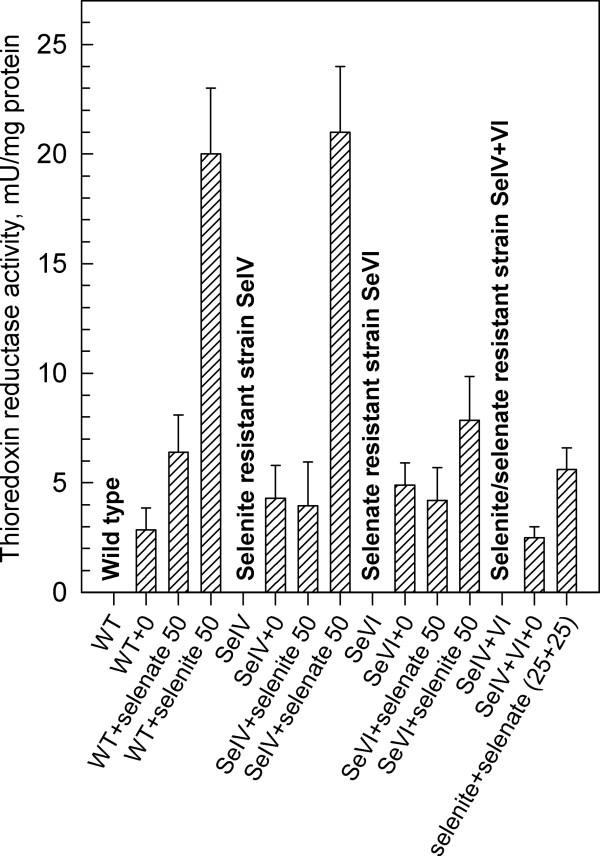
**Activity of thioredoxine reductase in asynchronous cultures of *Scenedesmus quadricauda***. Activity of thioredoxine reductase in asynchronous cultures of the wild type and selenium resistant strains of *Scenedesmus quadricauda *grown at the concentrations of selenite or selenate (0 and 50 mg Se × l^-1^): WT: wild type; SeIV: selenite resistant strain; SeVI: selenate resistant strain; SeIV+VI: selenite/selenate resistant strain. Samples were collected after 12 hours of cultivation. A specific activity of the TR was expressed as units per mg of cell proteins, where a unit is defined as the amount of enzyme that will cause an absorbance change of 1 at 412 nm using 200 μM NADPH per min. Data are presented as means ± S.D. of triplicate experiments.

We have measured the activity of thioredoxin reductase (TR) of *S. quadricauda *in both wild type and strains resistant to selenite (SeIV) or selenate (SeVI) or both compounds (SeIV+VI). Asynchronous cultures were grown in the presence (50 mg Se × l^-1^) and absence of Se added as selenite or selenate or a mixture of both compounds (Figure 5). In the wild type, the TR activity increased markedly at the concentration of 50 mg Se × l^-1 ^of selenium. The activity was higher when Se was added as selenate (20 mU × mg^-1^) than as selenite (6 mU × mg^-1^). In selenite resistant strain, SeIV at a concentration of selenite 50 mg Se × l^-1^, the TR activity was comparable to the activity in control cells grown without selenium. In the presence of selenate, the TR activity, however, increased rapidly (21 mU × mg^-1^). In the selenate resistant strain SeVI, the TR activity was again higher when cultivated with the more toxic Se form for a given strain – selenite in this case (8 mU × mg^-1^). In the strain SeIV+VI resistant to both selenite and selenate, the TR activity in the presence of both forms of Se (25+25 mg Se × l^-1 ^respectively) was low and comparable to untreated cells (6 mU × mg^-1^).

**Figure 6:**
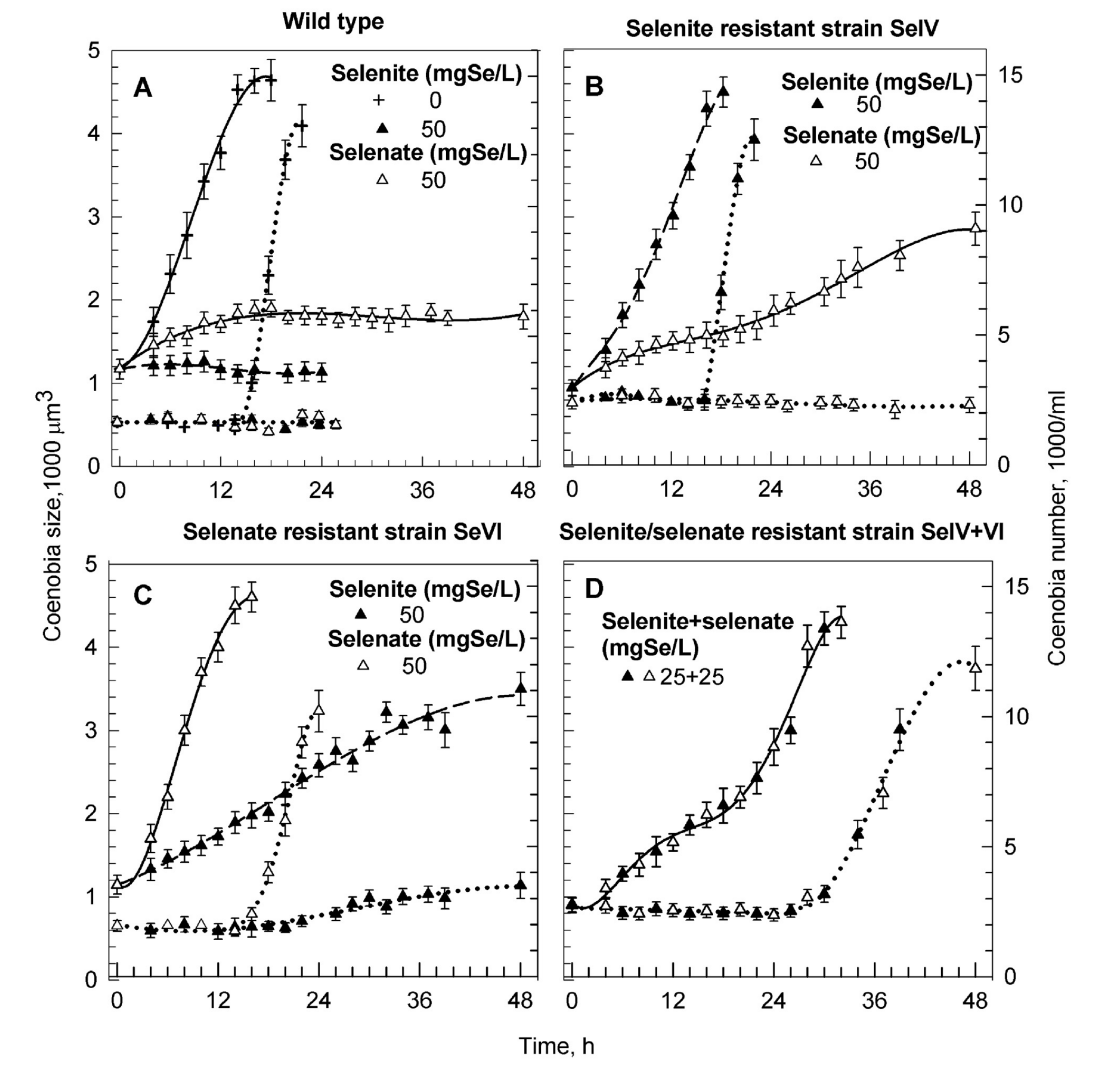
**Changes in coenobia size and coenobia number during the cell cycle of synchronous cultures of *Scenedesmus quadricauda***. Changes in coenobia size (solid lines) and coenobia number (dotted lines) during the cell cycle of synchronous cultures of wild type (**A**), selenite resistant (**B**), selenate resistant (**C**) and selenite+selenate resistant (**D**) strains of *Scenedesmus quadricauda *grown in the presence of 50 mg Se × l^-1 ^of selenite or selenate or selenite+selenate. Data are presented as means ± S.D. of triplicate experiments.

**Figure 7:**
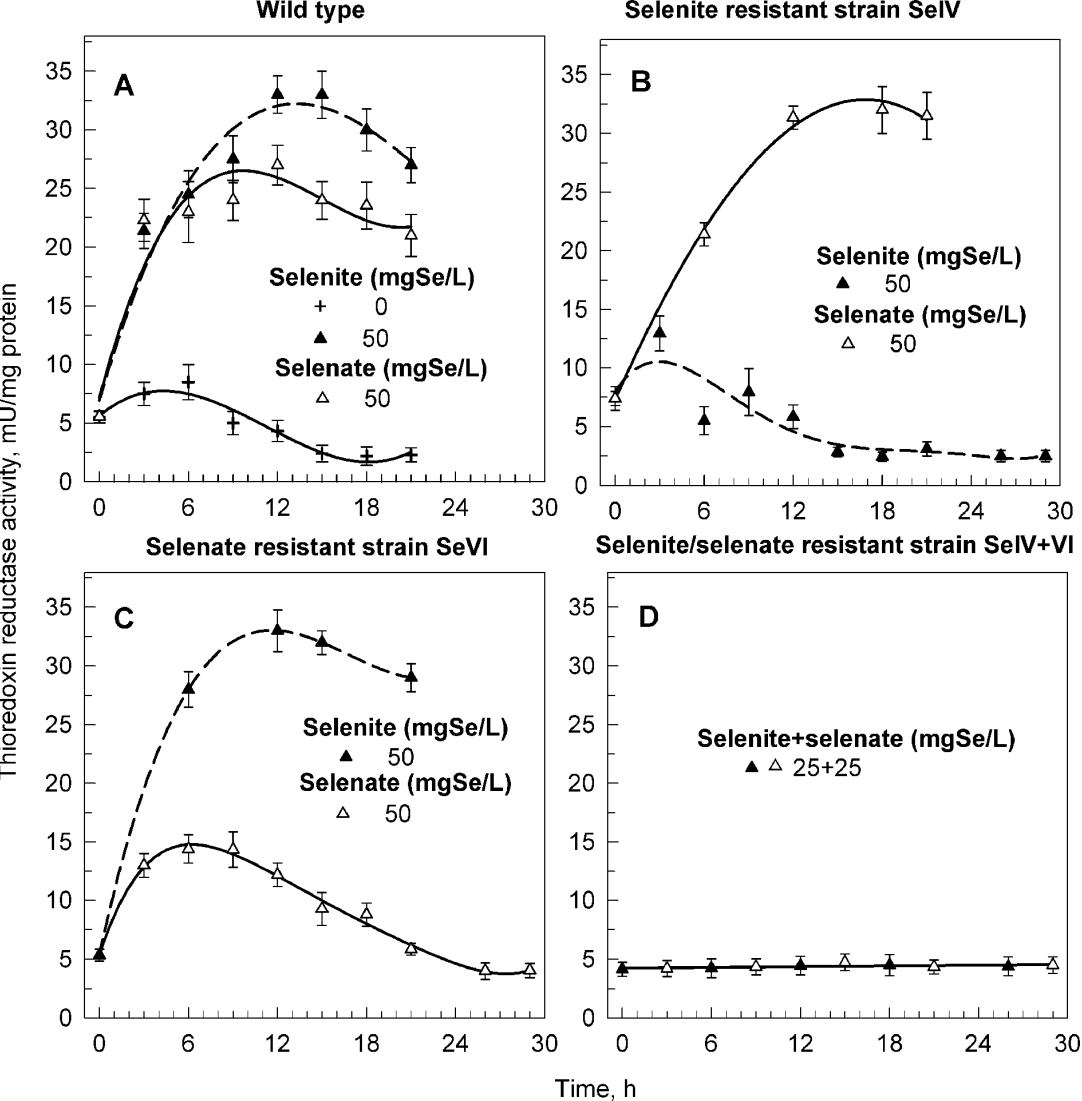
**Activity of thioredoxine reductase during the cell cycle in synchronous cultures of *Scenedesmus quadricauda***. Activity of thioredoxine reductase during the cell cycle in synchronous cultures of the wild type (**A**), selenite resistant (**B**), selenate resistant (**C**) and selenite/selenate resistant (**D**) strains of *Scenedesmus quadricauda *grown in the presence of 50 mg Se × l^-1 ^of selenite or selenate or selenite+selenate. A specific activity of the TR is expressed as units per mg of cell proteins, where a unit is defined as the amount of enzyme that will cause an absorbance change of 1 at 412 nm using 200 μm NADPH per minute. Data are presented as means ± S.D. of triplicate experiments.

All strains studied were also followed in synchronized cultures and the cell number and mean cell volume (Figure 6) were monitored during the cell cycle together with TR activity (Figure 7). Cells of the wild type grew poorly at 50 mg Se × l^-1 ^Se if added as selenate and died when Se was added as selenite. They did not divide with any of the Se forms (Figure 6A, triangles). Compare with the untreated culture (Fig 6A, crosses). Selenite resistant strain SeIV grew normally at 50 mg Se × l^-1 ^selenite and divided correspondingly to the untreated wild type (compare Figure 6A and 6B). When Se was added as selenate, the growth rate was slowed and no division occurred (Figure 6B). Selenate resistant strain SeVI grew normally at 50 mg Se × l^-1 ^of selenate and slowly at 50 mg Se × l^-1 ^of selenite (Figure 6C). Interestingly, at least some of the cells were able to divide in the presence of selenite though the division was delayed (Figure 6C). The strain SeIV+VI resistant to both selenite and selenate cultured in a mixture of selenite and selenate (25+25 mg Se × l^-1 ^respectively) grew slowly, but cells reached normal size. They had a long cell cycle (48 hr) and started to divide at the 30^th ^hour (Figure 6D).

The initial TR activity in both wild type and resistant strains was the same at the beginning of the cell cycle (about 5 mU × mg^-1^). During the growth phase of the untreated wild type, the activity increased slightly and then declined gradually to a constant low level (Figure 7A, crosses). A similar pattern was observed also in resistant strains SeIV and SeVI, if grown in the presence of selenium compound(s) to which they were resistant (Figures 7B and 7C). In the wild type cultivated with 50 mg Se × l^-1 ^as selenite or selenate, the activity increased extensively during the growth phase (up to 32 and 26 mU × mg^-1 ^respectively) and persisted at a high level till the end of the cell cycle. The TR activity was higher in the presence of selenite than in the presence of selenate (Figure 7A). Similarly the TR activity increased in the strains SeIV and SeVI when grown in the presence of Se compounds, to which they were not resistant (33 mU × mg^-1^) (Figures 7B and 7C). In the case of strain SeIV+VI the TR activity was low (about 5 mU × mg-1) during the whole cell cycle (Figure 7D).

The present results indicate that the activity of thioredoxin reductase is affected by selenium treatment in both a dose-dependent and toxic-dependent manner. The more toxic the selenium forms for the given algal strain are, the higher the TR activity present. This indicates that the activity of TR in algal cells is a reaction to the toxic effect of selenium. This is in agreement with findings in mammalian cells, where increased resistance to selenium cytotoxicity in cells with high levels of active TR, was demonstrated [27]. The authors concluded that a high level of active TR or a capacity to respond by inducing the expression of TR is a crucial mechanism for cells to survive exposure to sub-toxic/toxic levels of selenium compounds. TR over-expressing cells, which were preincubated for 72 h with 0.1 μM selenite, were significantly more resistant to selenite cytotoxicity than control cells [27].

TR is assumed to be an important enzyme in protecting against selenium cytotoxicity. The enzyme may protect cells against selenium cytotoxicity by at least three different mechanisms [27]. One mechanism is the direct reduction and detoxification of hydroperoxides including lipid-hydroperoxides and hydrogen peroxide [51]. The second mechanism involves reduction of thioredoxin and regeneration of antioxidants like ubiquinone [52]. The third and maybe most important mechanism is restoration of intracellular thiols lost by oxidation and also reduction of selenite to elemental selenium with a comparably low toxicity [53].

Concerning the present results, the TR activity increased in the presence of toxic levels of selenium as it was found in mammalian cells. This would indicate a defensive response of algal cells to selenium toxicity but it can be also only a general reaction to stress without a direct relation to selenium.

## Conclusion

Selenium toxicity in the wild type cells of the green alga *Scenedesmus quadricauda *increased with increasing dosage of selenium added as selenite or selenate. The selenium compounds caused cell growth inhibition as well as a block of cell division. Both of the compounds caused dose dependent accumulation of selenomethionine (SeMet), an organic form of selenium. Of the two compounds, selenite was more toxic than selenate. This was probably due to an increase of a selenomethionine (29% of SeMet in the case of selenate and 41% of SeMet in the case of selenite). The increasing toxicity was also accompanied by an increase in thioredoxin reductase (TR) activity implying a role for it in the stress response. Selenium toxicity increased in cultures grown under sulfur deficient conditions, indicating interference of selenium with sulfur metabolism. However, the total selenium content in biomass was proportional to selenium concentration in nutrient solution and independent of sulfate concentration.

We selected three strains resistant to high concentrations of different selenium compounds. The strains differed in the compound(s) to which they were resistant as well as in the degree of the resistance. The selected strains were resistant to selenite or selenate while still sensitive to the other compound. The strain resistant to combinations of both selenite and selenate showed the lowest resistance of all selected strains. This indicates that modes of action of selenite and selenate are different and modification of a common pathway for both compounds can provide only a limited degree of resistance. The selenite resistant strain (SeIV) showed very low levels of total selenium and its organic form selenomethionine if treated with selenite, implying that its resistance is caused by exclusion, probably due to downregulation of a sulfate transporter. Since its level of total selenium and selenomethionine are similar to wild type levels if treated by selenate the import mechanism for selenite and selenate seem to be different. On the contrary, the selenate resistant strain (SeVI) had the same levels of both total selenium and selenomethionine in the presence of selenate. This indicates that the mechanism of resistance is not due to changes in the import level but rather to some unknown internal mechanism decreasing the selenium toxicity. Interestingly, to gain resistance to both selenate and selenite the cells probably modified the mechanism responsible for the conversion of selenium into its organic compound, selenomethionine. Therefore, it appears that there are at least three different and independent mechanisms able to establish resistance to selenium compounds.

In wild type and all the resistant strains the addition of a toxic form of selenium for a particular strain was accompanied with an increase in the activity of thioredoxin reductase (TR). The TR activity was affected in dose-dependent and toxic-dependent manner. The more toxic the selenium form for the given algal strain, the higher the TR activity found. This indicates that TR activity is either one of the hallmarks of stress caused by selenium (or general stress) and/or, more appealingly, it is actively involved in detoxification of selenium as indicated in the literature.

The study provides a new insight into the impact of selenium on green algae with reference to its toxicity and bioaccumulation. Selenium is an essential micronutrient in the diet of many organisms, including humans and significant health benefits have been attributed to it. Selenomethionine is, due to its enhanced bioavailability, essential both in biomedicine and to complement the diet of domestic animals. The enrichment of the selenate resistant strains in selenomethionine could be scaled up to produce selenium enriched algal biomass. Also, the selected selenium resistant strains could be used for bioremediation of selenium-contaminated surroundings.

## Methods

### Experimental organism, culture growth conditions

The chlorococcal alga *Scenedesmus quadricauda *(TURP.) BRÉB. Strain Greifswald/15 was obtained from the Culture Collection of Autotrophic Microorganisms (Institute of Botany, Třeboň, Czech Republic). The species belongs the algae, which are able to divide by multiple fission into more than two daughter cells connected in coenobia. Actually 2-, 4-, or 8-celled coenobia can be formed. The cells are firmly connected in coenobium for the whole cell cycle. Marginal cells of the coenobium (not inner ones) are ornamented by two projecting spines, which are a part of the cell wall consisting of sporopollenin and are typical for the species. Cultures of *S. quadricauda *were cultivated at 30°C in liquid mineral medium [54] in a laboratory-scale photobioreactor. The cultures were aerated with air containing 2% carbon dioxide (v/v). The photobioreactor was illuminated from one side by fluorescent lamps (Osram DULUX L, 55 W/840, Italy) at an incident radiance of 100 W × m^-1 ^(400–720 nm) at the surface. To obtain synchronized cells, the cultures grown at alternating light and dark periods (14:10 h).

### Selenium treatment

The selenium was added as selenite or selenate in the range of concentrations (5 – 400 mg Se × l^-1^) to nutrient medium at the beginning of cultivation. Three replicate samples were used for all analyses and measurements. The average value was used for the construction of graphs. Standard deviations were indicated as bi-directional bars.

### Determination of total Se content (ICP-MS)

Nitric acid (65%, p.a., Merck Darmstadt, Germany) and hydrogen peroxide (30%, Analpure, Analytika Prague, Czech Republic) were used in the mixture used to digest biomass for the determination of total Se. A sample (0.1 g) of biomass was digested with 4 ml of nitric acid and 2 ml of hydrogen peroxide at 190°C in a PTFE vessel in a closed microwave digestion system (Berghof, Germany). After evaporation of excess acid in the same MW system, the resulting solution was transferred to a volumetric flask (100 ml) and filled with water (18.2 MΩ resistivity, Millipore Simplicity, Bedford, MA, USA).

An Inductively coupled plasma – mass spectrometer Agilent 7500ce (Agilent Technologies, Japan) was used for analysis of sample solutions. For quantification of Se, a standard addition method was used to eliminate matrix effects of residual carbon and other matrix elements. Se isotopes 77 and 82 were used, as these isotopes did not suffer from Ar-based spectral interferences. All data are presented as means ± S.D. of five experiments.

### Determination of SeMet content (ICP-MS)

Methanesulfonic acid hydrolysis of proteins in biomass was applied in the determination of total SeMet content in biomass according to[55]. 100 mg of algal biomass (dry weight) was mixed with 10 ml of methanesulfonic acid (4 mol × l^-1^, Sigma-Aldrich, Prague, Czech Republic) and 0.2 ml 2-mercaptoethanol (Fluka, Prague, Czech Republic) and refluxed for 16 hours. The resulting solution was filled to 100 ml with deionized water and filtered through a 0.45-μm syringe filter (regenerated cellulose) prior to chromatographic analysis.

For the separation of Se species, anion-exchange chromatography with ICP-MS detection was applied. A strongly basic anion exchange column Hamilton PRP-X100 (4.6 × 150 mm + 4.6 × 25 mm guard column, Hamilton Company, Nevada, USA) was operated in isocratic mode with ammonium acetate/methanol mobile phase [pH 5.0, 40 mM, 1% v/v methanol, 0.6 ml × min^-1^] at 25°C. Se species were detected using Se isotopes 77 and 82. Selenomethionine (> 99%, Sigma-Aldrich, Prague, Czech Republic) standard solutions in methanesulfonic acid were used for calibration. All data are presented as means ± S.D. of five experiments.

### Enzyme activity assay

Thioredoxin reductase (TR) activity was determined by the method according to (Holmgren and Bjőrnstedt, 1995). Cells were centrifuged at 4000 rpm for 5 minutes, washed with buffer A [50 mM Tris/HCl, 1 mM EDTA, pH 7.5] and disintegrated by vortexing with zircon beads (diameter 0.7 μm, Biospec, Bartlesville, OK, USA) 2:1 in buffer A with plant protease inhibitors (Sigma-Aldrich, Prague, Czech Republic) for 6 minutes. The extract was centrifuged at 13 000 rpm for 15 minutes and the supernatant frozen in liquid nitrogen. Cell extract (10 μl) was mixed with 490 μl of buffer A containing 2 μM Trx (*E. coli*, Sigma-Aldrich, Prague, Czech Republic), 500 μg × ml^-1 ^insulin (bovine pancreas, Sigma-Aldrich, Prague, Czech Republic) and 200 μM NADPH (tetrasodium salt, Calbiochem, San Diego, CA, USA). The mixture was incubated at 37°C for 20 minutes. Reaction was terminated by addition of 500 μl of 6 M guanidine hydrochloride (Sigma-Aldrich, Prague, Czech Republic) containing 1 mM 5,5'-dithiobis (2-nitrobenzoic acid) (DTNB, Sigma-Aldrich, Prague, Czech Republic). The increase in spectrophotometric absorbance at 412 nm was read from a microtitre plate using an Infinite F200 spectrophotometer (TECAN, Mannendorf, Switzerland). Reaction without cell extract and reaction with pure TR (*E. coli*, Calbiochem, San Diego, CA, USA) in place of cell extract were used as negative and positive controls, respectively. Specific activity of the enzyme was expressed as units per mg protein, where 1 unit is defined as the amount of enzyme that will cause an absorbance change of 1 at 415 nm using 200 μM NADPH per min. Total cell protein concentration was determined using Bradford methods [56]. All data are presented as means ± S.D. of triplicate experiments.

### Cell size and number measurements

Cells were immediately fixed by glutaraldehyde (2% v/v). Fixed cells with densities ranging from 1 × 10^6 ^to 1 × 10^7 ^cells × ml^-1 ^were diluted in 10 ml electrolyte solution [0.9% NaCl]; cell concentrations and cell size distributions were determined using a Coulter Multisizer III (Coulter Corporation, Florida, USA).

### Microphotography

Observations in transmitted light and fluorescence microscopy were carried out using a BX51 microscope (Olympus, Japan) equipped with DIC (Differential interference contrast) and a U-MWIG2 filter block (excitation 520 – 550 nm, emission 580 nm). The microphotographs were taken using a CCD camera (F-View II).

## Authors' contributions

DU and MV developed the experimental design, conducted and carried out the majority of the experiments and drafted the manuscript. ID selected resistant strains and with JM performed chemical analyses. KB and MC participated in cell cycle studies. MH carried out the enzyme assays. JD and VZ conceived of the study and participated in its design and coordination. All authors read and approved the final manuscript.
